# Effects of dietary supplementation of arginine-silicate-inositol complex on absorption and metabolism of calcium of laying hens

**DOI:** 10.1371/journal.pone.0189329

**Published:** 2018-01-23

**Authors:** Kazim Sahin, Cemal Orhan, Mehmet Tuzcu, Armagan Hayirli, James R. Komorowski, Nurhan Sahin

**Affiliations:** 1 Department of Animal Nutrition and Nutritional Disorders, Faculty of Veterinary Medicine, Firat University, Elazig, Turkey; 2 Division of Biology, Faculty of Science, Firat University, Elazig, Turkey; 3 Department of Animal Nutrition and Nutritional Disorders, Faculty of Veterinary Medicine, Atatürk University, Erzurum, Turkey; 4 Scientific and Regulatory Affairs, Nutrition 21 Inc, New York, United States of America; Gaziosmanpasa University, TURKEY

## Abstract

The effects of supplementation of arginine-silicate-inositol complex (ASI; 49.5–8.2–25 g/kg, respectively) to laying hens were investigated with respect to eggshell quality, calcium (Ca) balance, and expression of duodenal proteins related to Ca metabolism (calbindin and tight junction proteins). A total of 360 laying hens, 25 weeks old, were divided into 3 groups consisting of 6 replicate of cages, 20 birds per cage. The groups were fed a basal diet and the basal diet supplemented with 500 or 1000 mg ASI complex per kilogram for 90 days. Data were analyzed by ANCOVA using data during the first week of the adaptation period as covariates. As the ASI complex supplementation level increased, there were increases in feed intake (*P <* 0.0001), egg production (*P <* 0.001), egg weight (*P <* 0.0001) and eggshell weight (*P <* 0.001) weight, and shell thickness (*P <* 0.001) and decreases in feed conversion ratio and cracked egg percentage (*P <* 0.0001 for both). Concentrations of serum osteocalcin (*P <* 0.0001), vitamin D (*P <* 0.0001), calcium (*P <* 0.001), phosphorus (*P <* 0.001), and alkaline phosphatase (*P <* 0.008) as well as amounts of calcium retention (*P <* 0.0001) and eggshell calcium deposition (*P <* 0.001), and Ca balance (*P <* 0.0001) increased, whereas amount of calcium excretion (*P <* 0.001) decreased linearly in a dose-dependent manner. The ASI complex supplementation increased expressions of calcium transporters (calbindin-D28k, N sodium-calcium exchanger, plasma membrane calcium ATPase, and vitamin D receptor) and tight junction proteins (zonula occludens-1 and occludin) in the duodenum in a linear fashion (*P* < 0.0001 for all). In conclusion, provision of dietary ASI complex to laying hens during the peak laying period improved eggshell quality through improving calcium utilization as reflected by upregulation of genes related to the calcium metabolism. Further studies are needed to elucidate the contribution of each of the ASI complex ingredients.

## Introduction

The eggshell quality is one of the fundamental economic interests for the egg industry to maximize product durability during collection, storage, and transportation. Annual loss due to reduced marketing value of eggs associated with cracked and/or broken eggshells was estimated to be about 247 million US dollars in the USA [1]. Approximately 6–8% of the total egg production is not usable and/or marketable due to the poor shell quality [2]. At the grand-parent and parent stocks as well as hatchery unit, it is well known that the effective growth of a chicken embryo depends on the integrity of the eggshell. Calcification promotes eggshell stiffness, which does not allow internalization of pathogens from the outside and eliminates water loss from inside the egg. Moreover, eggshell is a sufficient calcium source for forming and strengthening the skeleton of the embryo [3, 4].

Many factors influence the eggshell quality including genetic basis, laying hen age, nutrition, and environmental conditions [4–6]. Calcium is the predominant mineral related to external and internal egg quality. Intestinal absorption of dietary calcium has a central role in the regulation of calcium homeostasis [7]. Ionized calcium is absorbed mostly in the small intestine, accounting for approximately 90% of overall calcium absorption [8]. The transcellular (active) and paracellular (passive) pathways involved in intestinal calcium absorption. Calcium transport proteins [*i*.*e*., calbindin-D9k/-D28k (CaBP-28k), N sodium-calcium exchanger-1 (NCX-1), and plasma membrane calcium ATPase (PMCA1)] are employed in calcium reabsorption through active transport [6]. NCX1 and PMCA1 regulate intracellular calcium ion excretion via exchanging outer sodium ions for inner calcium ions [9] and facilitating calcium ion excretion [10], respectively. Tight junction proteins [*i*.*e*., zonula occludens-1 (ZO-1), occludin (OCLN), and claudin (CLDN) are regarded as essential in calcium absorption through passive transport [11]. ZO-1 interacts with the actin cytoskeleton [12, 13]. OCLN is a transmembrane protein sealing intercellular junctions [14]. CaBP-9k and CaBP-28k involve in buffering intracellular calcium concentrations in the kidney and duodenum, respectively [15].

Major nutrients that affect the eggshell quality are calcium, phosphorus, and vitamin D_3_ [16–18]. Arginine-silicate-inositol complex (ASI; 49.5–8.2–25 g/kg) has been formulated to augment calcium metabolism. It also contributes to vascular and bone health [19, 20]. The silicon and inositol in the complex increase arginine bioavailability [21]. Our research group has previously reported the positive effects of the ASI complex on egg production, egg external quality, and bone metabolism in heat-stressed quails [20, 22]. The ASI complex may augment expressions of intestinal proteins to calcium metabolism. The experiment was therefore performed to evaluate alterations in metabolic profile along with the expressions of calcium transport proteins [CaBP-28k, (NCX)-1, PMCA1, and vitamin D receptor (VDR)] and tight junction proteins (ZO-1 and OCLN) in response to dietary ASI supplementation during the peak phase in laying hens.

## Materials and methods

### Animals, management, diets, and experimental design

Three hundred and sixty (25-week old) white laying hens (Lohman LSL) cared in accordance with animal welfare regulations in a commercial farm. The experimental protocol with respect to management, handling, and sampling was approved by the Animal Ethics Committee of Veterinary Control and Research Institute of Elazig, Turkey. The birds were housed in wire cages and exposed to a 16L:8D illumination cycle. Feed and fresh water were offered *ad libitum* throughout the experiment. After a 7-day adaptation period, hens were assigned randomly to one of three groups: *ad libitum* consumption of basal diet (Table 1) formulated to meet nutrient requirement [23] and supplementation with 0, 500 or 1000 mg the ASI complex (Nitrosigine^®^, Nutrition 21 Inc, Purchase, NY) per kilogram diet through adding 7 kg newly reconstructed vitamin-mineral premix per tonne feed. The ASI complex contained arginine, silicone, and inositol at amounts of 49.5, 8.2, and 25 g per kilogram, and 917.3 g corn starch being a carrier. The basal premix (6 kg) was added with 1 kg cornstarch (0 mg/kg ASI Group), 0.5 kg corn starch plus 0.5 kg ASI complex (500 mg/kg ASI Group), or 1 kg ASI complex (1000 mg/kg ASI Group) to establish the experimental groups. Thus, dosage was projected to deliver 0, 55, and 110 mg ASI complex per day at the assumption of feed intake being 110 g/d.

**Table 1:** Ingredient and nutrient composition of the basal diet.

Ingredient	g/kg
Corn	617.1
Soybean meal	259.4
Soy oil	20.0
Limestone	90.0
Dicalcium phosphate	2.5
Vitamin-mineral premix^a^	7.0
Sodium chloride	2.0
Sodium bicarbonate	2.0
**Chemical analyses**^b^	
Metabolisable energy (MJ/kg)	11.60
Crude protein	167.2
Crude fat	46.0
Crude fiber	36.4
Crude ash	125.1
Calcium	40.0
Phosphorus	33.0
Methionine	4.2
Lysine	8.6

^a^Supplied per kg diet: retinyl acetate, 41.28 mg; cholecalciferol, 60 μg; dl-α-tocopheryl acetate, 30 mg; menadione sodium bisulfite, 2,5 mg; thiamine-hydrochloride, 3 mg; riboflavin, 7 mg; niacin, 40 mg; d-pantothenic acid, 8 mg; pyridoxine hydrochloride, 4 mg; vitamin B_12_, 0.015 mg; vitamin C, 50 mg; folic acid, 1 mg; D-biotin, 0.045 mg; choline chloride, 125 mg; Mn (MnSO_4_-H_2_O), 80 mg; Fe (FeSO_4_-7H_2_O), 30 mg; Zn (ZnO), 60 mg; Cu (CuSO_4_-5H_2_O), 5 mg; Co (CoCl_2_-6H_2_O), 0,1 mg; I (KI), 0.4 mg; and Se (Na_2_SeO_3_), 0.15 mg.

The ASI complex contained arginine, silicone, and inositol at amounts of 49.5, 8.2, and 25 g per kilogram, corn starch (917.3 g) being a carrier. The basal premix (6 kg) was reconstituted by adding 1 kg corn starch, 0.5 kg corn starch plus 0.5 kg ASI complex, or 1 kg ASI complex to establish the experimental groups containing 0, 500, and 1000 mg ASI per kg diet.

^b^ Metabolisable energy, methionine and lysine are calculated based on tabular values for feedstuffs (Jurgens, 1996).

The experimental period was 90 days and each treatment group consisted of six replicate cages, each cage housing 20 birds. Experimental diets (Table 1) were stored in black plastic containers at 4°C to avoid photooxidation. Feed consumption was measured weekly and egg yield and egg weight were recorded daily.

### Calcium excretion, calcium retention, and balance study

At the last week of the experiment (82 d), 12 chickens from each group (two per replicate cage) were placed into individual battery cages (total n = 36) for determination of calcium balance. They were maintained on the same experimental diets as before. After 3 d of preliminary feeding, hens were offered a weighed amount of feed at 08:00 every day for the trial period. Total excreta was collected at the end of the day and pooled for 5 d, and then dried at 65°C for 5 d before retaining. Daily feed intake was calculated after deducting the residue from the feed offered. Daily apparent calcium retention was defined as dietary calcium not appearing in the excreta. The egg was collected daily for eggshell calcium content. Calcium balance was expressed as the difference between calcium retention and calcium deposited in the eggshell.

### Sample and data collection

At the end of the study, egg quality parameters (egg weight, shell weight, shell thickness, and Haugh unit) were measured on twelve eggs collected randomly from each of six replicate cages per experimental group. Haugh unit was calculated using following formula: Haugh unit = 100 x log (H + 7.57–1.7 x W^0.37^), where H = albumen height, mm and W = egg weight, g [24] after determining albumen height by a micrometer (Saginomiya, TLM-N1010, Tokyo, Japan) and egg weight.

For serum and tissue analyses, 12 chickens from each group (two per replicate cage) were sacrificed by cervical dislocation at the end of the study. The blood was drawn from the axillary vein using sterilized needles and syringes into test tubes. Serum was separated and centrifuged at 3,000 *g* for 10 min at 20°C, and then stored at -80°C until laboratory analyses. Duodenal tissues were rapidly excised, washed in cold sterile saline, and immediately stored at -80°C until analyses of proteins (calbindin D28k, OCLN, NCX-1, ZO-1, PMCA1, and VDR).

### Laboratory analyses

Feed samples were analyzed for crude protein (#988.05), ether extract (#932.06), crude fiber (#962.09), crude ash (#936.07), Ca (#968.08) and P (#965.17) [25]. Energy and amino acid (methionine and lysine) contents were calculated from tabular values of feedstuffs for poultry [26].

Diet, serum, excreta and eggshell calcium contents were determined using an atomic absorption spectrophotometer (AAS, Perkin-Elmer, Analyst 800, Norwalk, CT) as described by Ruuskanen et al. [27]. Serum phosphorus contents were determined using enzymatic assay as described by Pearson [28]. Serum alkaline phosphatase (ALP) concentration was measured using biochemical analyzer (Samsung LABGEO^PT10^, Seoul, Korea). Serum concentration of 25(OH)D_3_ was determined using an enzyme-linked immunosorbent assay kit (ALPCO Diagnostic^™^ Immunoassay Kits, Catalog #: 38-KIP-1921, Salem, NH) [29]. The absorbance was read at 450 nm using a microplate reader (EL800, BioTek, Winooski, VT). The intra-assay CV and inter-assay CV were 4.5% and 6.4% respectively. Serum osteocalcin (OC) was assessed by a commercially available enzyme-linked immunosorbent assay kit (ELISA; Chicken Osteocalcin ELISA Kit; LifeSpan BioSciences, Inc. Seattle, WA). The detection range was 1.56–100 pg/ml and sensitivity was 0.67 pg/ml. The intra-assay CV and inter-assay CV were 4.8% and 6.7%, respectively.

Protein profile and expression in duodenal (calbindin D28k, NCX-1, PMCA1, VDR, OCLN, ZO-1) samples were attained by western blot analysis. Exactly weighed samples were homogenized in 1:10 (w/v) in 10 m*M* Tris-HCl buffer at pH 7.4, comprising 0.1 m*M* NaCl, 0.1 m*M* phenylmethylsulfonyl fluoride, and 5 μ*M* soybean (soluble powder; Sigma, St. Louis, MO) as trypsin inhibitor. Tissue homogenate was centrifuged at 15,000 *g* at 4°C for 30 min and the supernatant was transferred into fresh tubes. Sodium dodecyl sulphate-polyacrylamide gel electrophoresis sample buffer containing 2% *β*-mercaptoethanol was added to the supernatant. Equal amounts of protein (20 μg) were electrophoresed and then transmitted to nitrocellulose membrane (Schleicher and Schuell Inc., Keene, NH). Nitrocellulose blots were washed twice for 5 min in phosphate buffered saline (PBS) and blocked with 1% bovine serum albumin in PBS for 1 h prior to the introduction of primary antibody. Chicken antibodies against calbindin D28k, OCLN, NCX-1, ZO-1, PMCA1, and VDR (Abcam, Cambridge, UK) were diluted (1:1000) in the same buffer containing 0.05% Tween-20. The nitrocellulose membrane was incubated overnight at 4°C with protein antibody. The blots were washed and incubated with horseradish peroxidase-conjugated goat anti-mouse IgG (Abcam). Specific binding was detected using diaminobenzidine and hydrogen peroxide as substrates. Protein loading was controlled using a monoclonal mouse antibody against β-actin antibody (Sigma). Samples were analyzed in quadruplicates and protein levels were determined densitometrically using an image analysis system (Image J; National Institute of Health, Bethesda, MD).

### Statistical analyses

A 10%-improvement in serum Ca concentration was considered to be significant at type I error of 0.05 with the power of 0.85 in sample size calculation. Data were subjected to ANCOVA using the PROC MIXED procedure [30]. Linear model to test effects of dietary ASI complex supplementation on animal performance and egg quality was *yij = μ + b*_*0*_ *+ Li + ej*, where *y* = response variable, *μ* = population mean, *b*_*0*_ = covariate, *L* = ASI supplementation level, and *e* = residual error being *N* (σ, μ; 0, 1). Egg production and egg weight data during the adaptation period were used as covariates for corresponding response variables. The model also included orthogonal and polynomial contrast to determine ASI supplementation effect and changes in response variables upon increasing dietary ASI supplementation. Moreover, Pearson’s correlation analysis was used to assess the correlation between eggshell quality, vitamin D, calcium metabolism, and calcium transport proteins’ expressions. Statistical significance was declared at *P* < 0.05.

## Results

**Fig 1:**
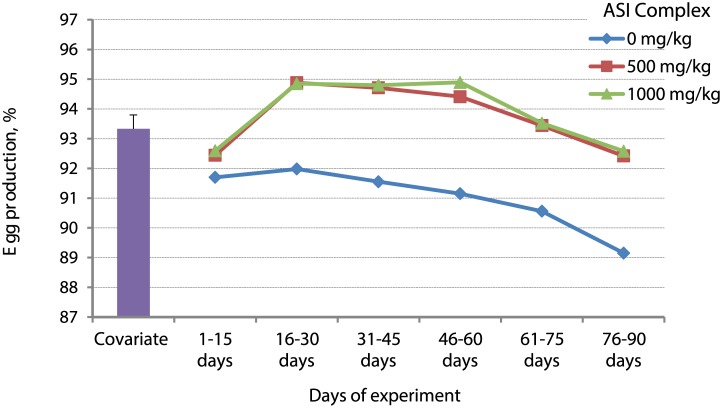
Effects of ASI (arginine-silicate-inositol) complex (♦, 0 mg/kg; ■, 500 mg/kg; ▲, 1000 mg/kg) supplementation on egg production. Covariate is average initial egg production of all chickens first 7 days prior to the experimental period at age of 25 weeks. Pooled SEM = 0.27.

Hens supplemented with the ASI complex had greater feed intake (112.67 *vs*. 109.82 g; *P* < 0.0001), higher egg production (93.80 *vs*. 91.02%; *P* < 0.001; Fig 1), and had better feed conversion efficiency (1.89 *vs*. 1.99; *P* < 0.001) than hens fed the control diet (Table 2). The ASI complex supplementation reduced defective egg percentage (0.80 *vs*. 1.18%; *P* < 0.0001), increased egg weight (63.76 *vs*. 60.53 g; *P* < 0.0001) and eggshell weight (9.30 *vs*. 7.66 g; *P* < 0.001), and thickened eggshell (0.39 *vs*. 0.31 mm; *P* < 0.001) as compared to chickens fed the control diet. These performance variables improved in linear fashion as dietary ASI complex supplementation increased (Table 2). Haugh unit was unaltered by the treatments.

**Table 2:** Effects of dietary ASI (arginine-silicate-inositol) complex supplementation on performance and egg quality^a^.

Variable	ASI, mg/kg			SEM	Statistical significance, *P* > F^b^		
	0	500	1000		S	L	Q
Feed intake, g/d	109.82	112.63	112.70	0.26	0.0001	0.0001	0.001
Egg production, %	91.02	93.72	93.87	0.45	0.001	0.0001	0.034
Feed conversion ratio^c^	1.99	1.89	1.88	0.01	0.0001	0.0001	0.027
Cracked broken egg, %	1.18	0.82	0.77	0.03	0.0001	0.0001	0.006
Egg weight, g	60.53	63.63	63.89	0.27	0.0001	0.0001	0.0001
Shell weight, g	7.66	9.13	9.47	0.17	0.001	0.001	0.009
Shell thickness, mm	0.31	0.39	0.38	0.01	0.001	0.001	0.001
Haugh unit^d^	92.43	92.15	92.58	0.55	0.877	0.863	0.633

^a^Data are the least square means over a 90-day animal experimentation. n = 6 cages, 20 birds per cage.

^b^Statistical contrast: S = ASI complex supplementation effect (hen supplemented with ASI complex vs. hen not supplemented with ASI complex); L = Linear effect of increasing dietary ASI complex; Q = Quadratic effect of increasing dietary ASI complex. SEM = Standard error of the mean.

^c^Feed conversion ratio = g feed consumed per g egg mass (egg production x egg weight).

^d^Haugh unit = 100 x log(*H* + 7.57–1.7 x *W*^0.37^) where *H* = albumen height, mm and *W* = egg weight, g.

The ASI complex supplementation positively affected serum parameters. Its supplementation up to 1000 mg/kg increased serum concentrations of OC, vitamin D, ALP, calcium, and phosphorus by 1.44 (*P* < 0.0001), 1.18 (*P* < 0.0001), 1.10 (*P* < 0.008), 1.35 (*P* < 0.001), and 1.38 (*P* < 0.001) folds in a linear fashion, respectively (Table 3). Hens supplemented with the ASI complex consumed higher amount of calcium (4.51 *vs*. 4.39 g; *P* < 0.0001), excreted lower amount of calcium (1.30 *vs*. 1.75 g; *P* < 0.001), retained higher amount of calcium (3.22 *vs*. 2.64 g or 71.3 *vs*. 60.1% of the consumption, *P* < 0.0001), and deposited higher amount of calcium in eggshell (2.44 *vs*. 2.11 g; *P* < 0.001). The ASI complex supplementation level linearly increased overall Ca balance (0.78 *vs*. 0.53 for the ASI complex supplemented vs. control; *P* < 0.001).

**Table 3:** Effects of dietary ASI (arginine-silicate-inositol) complex supplementation on serum parameters and calcium metabolism^a^.

Variable	ASI, mg/kg			SEM	Statistical significance, *P* > F^b^		
	0	500	1000		S	L	Q
*Serum*^c^							
Osteocalcin (ng/ml)	6.14	8.73	8.84	0.30	0.0001	0.0001	0.004
Vitamin D (pg/ml)	49.29	57.86	58.00	1.58	0.0001	0.0001	0.045
ALP (U/L)^d^	109.51	118.72	120.14	2.30	0.008	0.004	0.183
Calcium (mmol/L)	2.26	3.15	3.06	0.03	0.001	0.01	0.264
Phosphorus ((mmol/L)	1.96	2.64	2.71	0.02	0.001	0.001	0.138
*Calcium balance*^e^							
Ca intake (g/d)	4.39	4.51	4.51	0.01	0.0001	0.0001	0.0001
Ca excretion (g/d)	1.75	1.28	1.31	0.07	0.001	0.001	0.018
Ca retention (g/d)	2.64	3.23	3.20	0.08	0.0001	0.0001	0.008
Eggshell Ca (g/d)	2.11	2.43	2.45	0.05	0.001	0.0001	0.044
Ca balance (g/hen/d)	0.53	0.80	0.75	0.04	0.0001	0.001	0.006

^a^Data are the least square means over a 90-day animal experimentation.

^b^Statistical contrast: S = ASI complex supplementation effect (hen supplemented with ASI complex vs. hen not supplemented with ASI complex); L = Linear effect of increasing dietary ASI complex; Q = Quadratic effect of increasing dietary ASI complex. SEM = Standard error of the mean.

^c^n = 12 hens per group.

^d^Alkaline phosphatase.

^e^n = 12 eggs per group.

**Fig 2:**
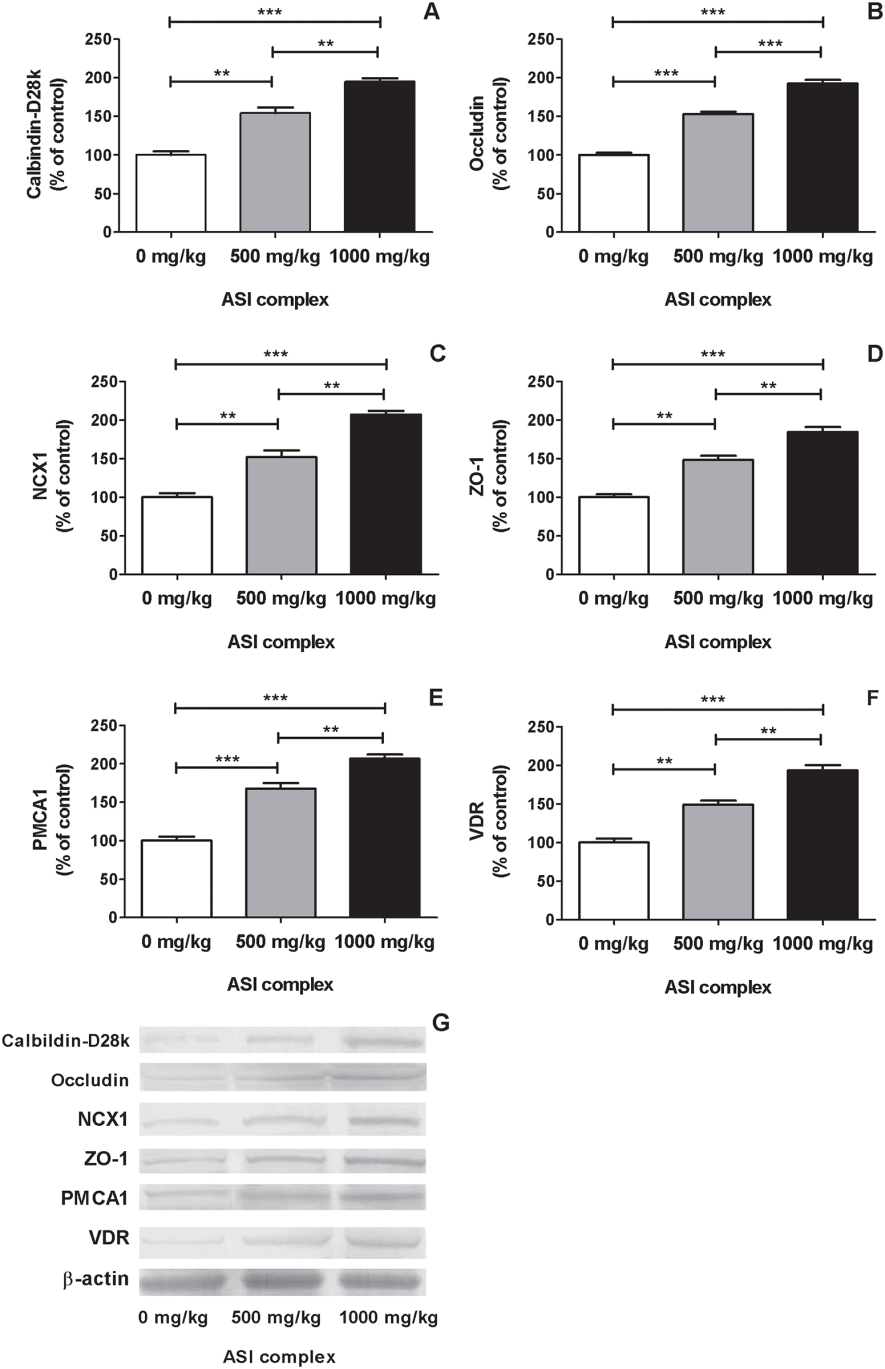
Effects of ASI (arginine-silicate-inositol) complex (0 mg/kg; 500 mg/kg; 1000 mg/kg) supplementation on the expression levels of the calcium transporters and tight junction proteins: calbindin-D28k (panel A), occludin (panel B), N sodium-calcium exchanger (NCX1, panel C), zonula occludens-1 (ZO-1, panel D), plasma membrane calcium ATPase (PMCA1, panel E), and vitamin D receptor (VDR, panel F) in duodenum. Tissue calbindin-D28k, occludin, NCX1, ZO-1, PMCA1, and VDR expressing levels (Panel G) western blot strips. Data are expressed as percent of control value. Each bar represents the mean and standard error of mean. Blots were repeated at least 3 times (n = 3). P<0.05; **P<0.01; ***P<0.001.

The effects of dietary ASI complex supplementation on expressions of the calcium transporters and tight junction proteins are shown in Fig 2. Hens supplemented with the ASI complex had greater expressions of CaBP-D28k (1.75-fold), OCLN (1.73-fold), ZO-1 (1.66-fold), NCX1 (1.80-fold), PMCA1 (1.87-fold), and VDR (1.71-fold) than hens not supplemented with the ASI complex (*P* < 0.0001 for all). Moreover, their expressions linearly increased up to 84 to 107% as the ASI complex supplementation was provided at 1000 mg/kg feed (*P* < 0.0001 for all).

Correlations among response variables were statistically significant (Table 4). There were weak positive correlations between shell thickness with serum vitamin D concentration (r = 0.595), and expressions of calcium retention (r = 0.549), calcium deposition in eggshell (r = 527), and expressions of CaBP-D28k (r = 0.472), OCLN (r = 0.555), NCX-1 (r = 0.530), ZO-1 (r = 0.546), PMCA1 (r = 0.562), and VDR (r = 0.517) and was a weak negative correlation between shell thickness and calcium excretion (r = -0.515). Defective egg percentage was weakly/moderately correlated with shell thickness (r = -0.516), serum vitamin D concentration (r = -0.632), calcium retention (r = -0.585), calcium deposition in eggshell (r = -0.581), and expressions of CaBP-D28k (r = -0.851), OCLN (r = -0.860), NCX-1 (r = -0.825), ZO-1 (r = -0.826), PMCA1 (r = -0.878), and VDR (r = -0.842) and correlated with calcium excretion (r = 0.530). Moreover, serum vitamin D concentration, calcium retention, and calcium deposition eggshell were positively correlated with expressions of Calcium transport proteins, whereas calcium excretion was negatively correlated with expressions of calcium transport proteins.

**Table 4:** Pearson’s correlation coefficients (r) among egg shell quality, vitamin D, calcium metabolism, and calcium transport proteins’ expressions.

	**Shell Thickness**	**Vitamin D**	**Ca excretion**	**Ca Retention**	**Eggshell Ca deposition**	**Calbindin D-28k**	**Occludin**	**NCX1**	**ZO-1**	**PMCA1**	**VDR**
**Cracked egg**	-0.516*	-0.632**	0.530*	-0.585*	-0.581*	-0.851**	-0.860**	-0.825**	-0.826**	-0.878**	-0.842**
**Shell thickness**		0.595**	-0.515*	0.549**	0.527*	0.472	0.555	0.530	0.546	0.562	0.517
**Vitamin D**			-0.482*	0.515*	0.438*	0.706*	0.705*	0.652	0.704*	0.670*	0.648
**Ca excretion**				-0.997**	-0.951**	-0.642	-0.614	-0.546	-0.689*	-0.559	-0.601
**Ca retention**					0.959**	0.708*	0.687*	0.616	0.754*	0.639	0.671*
**Eggshell Ca deposition**						0.646	0.664	0.571	0.754*	0.619	0.672*
**Calbindin D-28k**							0.969**	0.957**	0.961**	0.960**	0.966**
**Occludin**								0.990**	0.978**	0.986**	0.972**
**NCX1**									0.954**	0.970**	0.961**
**ZO-1**										0.948**	0.988**
**PMCA1**											0.944**

* Correlation is significant at the 0.05 level;

** Correlation is significant at the 0.01 level.

## Discussion

This experiment questioned if ASI supplementation to laying hens during the peak production period could improve eggshell quality, as reflected by reduced cracked egg percentage and increased eggshell thickness and eggshell calcium deposition, resulting from favoring more positive calcium balance via modulating expressions of calcium transporters and tight junction proteins. In this experiment, these parameters were limited to intestinal calcium metabolism. Indeed, kidney also plays an important role in calcium metabolism. This experiment also did not distinguish bone-specific ALP activity as well as free versus bound calcium concentrations in serum. These are pertinent to osteoblastic activity.

Dietary arginine and silicon influence calcium metabolism and bone mineralization in poultry [20, 31, 32]. Moreover, arginine is involved in both the synthesis of substrates (*e*.*g*, polyamine and L-proline) associated with collagen synthesis and the production of growth hormone, insulin-like growth factor-I, and nitric oxide [33, 34]. In agreement with our previous studies [20, 22], the ASI complex supplementation increased egg weight, shell thickness, shell weight during the peak laying period (Table 2). Additionally, serum OC and ALP levels, indicators of bone formation and calcium mobilization, increased linearly with dietary ASI complex supplementation during the peak laying period. Moreover, in response to increasing supplemental ASI complex level, increases in serum calcium and vitamin D concentrations, calcium retention and calcium deposition in eggshell, as well as a decrease in calcium excretion were accompanied by thickened eggshell (Tables 3 and 4). These suggest that ASI complex supplementation increases calcium and phosphorus availabilities and favors anabolism in calcium utilization. This postulation was also supported by up-regulations of calcium transport proteins (CaBP-D28k, OCLN, NCX1, ZO-1, and VDR) (Fig 2). In addition to calcium metabolism modulating effects [20, 35], arginine and silicon improve digestibility of dietary protein and macro minerals (*e*.*g*., calcium, phosphorus, and magnesium) and their retentions in bone under normal and heat stress conditions [22, 36].

Silicon [36] and arginine [35, 37] involve in calcium metabolism and are recommended in the alleviation of metabolic disturbance in calcium absorption, growth, dentition and ossification defects, rachitism, osteomalacia, decalcification, and convalescence. The ASI complex appears to act as an activator in calcium metabolism through up-regulating the duodenal CaBP-D28k, OCLN, NCX1, ZO-1, PMCA1, and VDR (Fig 2). These involve in the cation movement from the enterocytes towards the lamina propria [11], which could help increase calcium absorption to improve calcium balance (Table 3). Vitamin D also involves in intestinal absorption of calcium and acts as compensatory mechanism in regulating calcium metabolism [38]. Vitamin D increases tight-junction conductance and paracellular calcium transport [38], via regulating expressions of tight junction proteins [39]. Moreover, it activates protein kinase C that increases paracellular permeability [40]. The ASI complex supplementation increased serum vitamin D concentration and duodenal VDR expression, which may amplify its positive effect on calcium retention and excretion (Table 3). Vitamin D and its active metabolites (*i*.*e*., calcitriol) stimulate the CaBP-D28k expression [41]. There is an evolutionary adaptation for eggshell formation in avian animals. Calbindin is present in the intestine of birds before puberty and becomes abundant thereafter to cope with increased demand for calcium for eggshell formation [42]. Jiang et al. [15] reported that sodium bicarbonate supplementation increased CaBP-D28k expression in the duodenum, but not in kidneys, in chickens during the peak production period, suggesting that decreased urinary calcium excretion could be independent of duodenal CaBP-D28k expression. Vitamin D activates expression of the intestinal tight genes. This adaptation was ascertained by increased the duodenal NCX activity in response to feeding low-calcium diet [43].

In conclusion, this experiment has some major limitations. Our experiment dealt with the only effect of the ASI complex through intestinal route and did not measure bone-specific ALP activity as well as concentrations of ionized and bound calcium concentrations in bone. Its effect on calcium homeostasis through kidney cannot be neglected. Each component of the ASI complex is known to affect calcium metabolism. Our experiment cannot reveal their relative contribution to, synergistic and additive effects on calcium metabolism. Phosphorus intake and balance data would help explain some of responses. Protein:Energy intake and the ASI complex interaction effect on calcium metabolism would worth studying. In brief, providing the ASI complex to laying hens modulated calcium metabolism at the gene level resulting in reduced calcium excretion and increased calcium retention in body and calcium deposition in eggshell. The ASI complex supplementation enhanced eggshell quality as reflected by thickened eggshell and lowered cracked egg percentage during the peak period in laying hens.
